# Spottier Targets Are Less Attractive to Tabanid Flies: On the Tabanid-Repellency of Spotty Fur Patterns

**DOI:** 10.1371/journal.pone.0041138

**Published:** 2012-08-02

**Authors:** Miklos Blaho, Adam Egri, Lea Bahidszki, Gyorgy Kriska, Ramon Hegedus, Susanne Åkesson, Gabor Horvath

**Affiliations:** 1 Environmental Optics Laboratory, Department of Biological Physics, Physical Institute, Eötvös University, Budapest, Hungary; 2 Group for Methodology in Biology Teaching, Biological Institute, Eötvös University, Budapest, Hungary; 3 Danube Research Institute, Centre for Ecological Research, Hungarian Academy of Sciences, Vácrátót, Hungary; 4 Computer Vision and Robotics Group, University of Girona, Girona, Spain; 5 Department of Biology, Centre for Animal Movement Research, Lund University, Lund, Sweden; INRA-UPMC, France

## Abstract

During blood-sucking, female members of the family Tabanidae transmit pathogens of serious diseases and annoy their host animals so strongly that they cannot graze, thus the health of the hosts is drastically reduced. Consequently, a tabanid-resistant coat with appropriate brightness, colour and pattern is advantageous for the host. Spotty coats are widespread among mammals, especially in cattle (*Bos primigenius*). In field experiments we studied the influence of the size and number of spots on the attractiveness of test surfaces to tabanids that are attracted to linearly polarized light. We measured the reflection-polarization characteristics of living cattle, spotty cattle coats and the used test surfaces. We show here that the smaller and the more numerous the spots, the less attractive the target (host) is to tabanids. We demonstrate that the attractiveness of spotty patterns to tabanids is also reduced if the target exhibits spottiness only in the angle of polarization pattern, while being homogeneous grey with a constant high degree of polarization. Tabanid flies respond strongly to linearly polarized light, and we show that bright and dark parts of cattle coats reflect light with different degrees and angles of polarization that in combination with dark spots on a bright coat surface disrupt the attractiveness to tabanids. This could be one of the possible evolutionary benefits that explains why spotty coat patterns are so widespread in mammals, especially in ungulates, many species of which are tabanid hosts.

## Introduction

The coat pattern of cattle (*Bos primigenius*) has a remarkably large diversity ranging from homogeneous black and brown, through brown-white or black-white spotty, to homogeneous grey or white. These coat patterns are specific to species and races, and are the result of domestic breeding. The different coat patterns have some trivial advantages and disadvantages. The darkness of the coat influences the thermoregulation of the animal [1], [2], for example: black or brown coats absorb sunlight much more than white or grey ones. The visibility of the animal depends strongly on the brightness and colour of the coat in contrast to the background. At a given background the coat pattern also influences the visual detectability such that a spotty coat makes the animal conspicuous against a homogeneous background, but can endow with camouflaging at a structured background, for instance, similar to what has been shown by a classical experiment in the moth *Biston betularia* [3], [4]. During breeding the brightness, colour and spottiness of the coat in cattle and horses are usually of marginal importance and are the by-product of cross-breeding aiming to maximize other economically more important characteristics of the animal, e.g. the milk or meat production, weather-proofness, or the shape or size of the animals.

It has been demonstrated that the tabanid load of horses can be reduced by a homogeneously bright (white or grey) coat [5]. The study demonstrated that white horses attract much less blood-sucking female members of the family Tabanidae (tabanids henceforward) than dark (black or brown) horses. This phenomenon was partly explained by the polarizing capacity of the horse’s coat and the positive polarotaxis of tabanids. Tabanids lay their eggs onto plants or mud near water, and thus must find water for oviposition. Like aquatic insects in general [6], [7], [8], [9], [10], tabanids detect water by means of the horizontal polarization of light reflected from the water surface, thus they are attracted to horizontally polarized light [11]. The higher the degree of horizontal polarization of reflected light, the more polarotactic tabanids are attracted [12]. In [13] it was showed that female and male tabanids find water by the horizontally polarized water-reflected light, while the polarotaxis of female tabanids that serves host finding is independent of the angle of polarization, and both behaviour types are influenced by the degree of polarization.

Female tabanids suck blood from mammals to develop their eggs. During blood-sucking they transmit pathogens of different serious diseases [14], [15], [16]. Cattle can be so strongly annoyed by tabanid attacks that they cannot graze enough and consequently their milk and meat production is drastically reduced [17], [18]. Thus, a tabanid-resistant coat that has appropriate brightness, colour and pattern characteristics to minimize the attraction to tabanids is advantageous for the host animals of these blood-sucking flies.

Spotty coats are widespread among mammals [19]. We studied the influence of the size and number of spots on the attractiveness of targets to tabanids. In this work we report on the results of our field experiments using test surfaces with spots of different intensities and/or angles of polarization, the attractiveness of which to tabanids depends strongly on the spot size and number. Since the attraction of tabanids to their host animals is also likely determined by the polarizing capacity of the host’s coat [13], using imaging polarimetry, we measured the reflection-polarization characteristics of living cattle, spotty cattle coats and the test surfaces used in our experiments.

## Results

**Figure 1:**
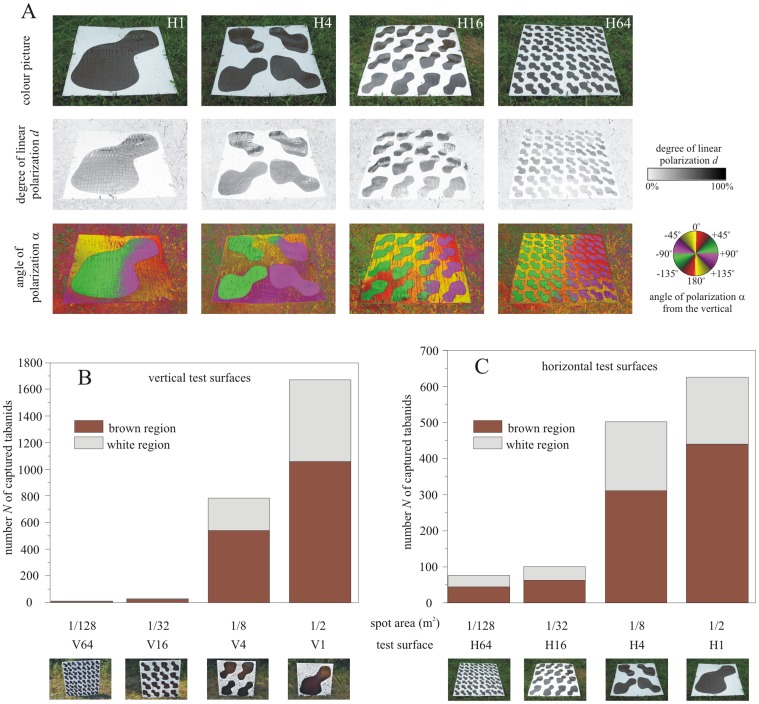
Results of experiments 1 and 2. (A) Colour pictures and patterns of the degree *d* and angle α (clockwise from the vertical) of linear polarization of light reflected from the shady brown-and-white spotty horizontal sticky test surfaces with 1 (H1), 4 (H4), 16 (H16) and 64 (H64) brown spots used in experiment 2 and measured by imaging polarimetry in the blue (450 nm) part of the spectrum when the optical axis of the polarimeter was −30° from the horizontal. (B, C) Number *N* of tabanids captured by the brown and white regions of the vertical and horizontal spotty and sticky test surfaces in experiments 1 and 2 as a function of the area (m^2^) covered by one brown spot.

On all test surfaces in experiments 1 and 2, the brown spots captured 1.4−3.7 times more tabanids than the white surface regions (Fig. 1, Supplementary Figs. S2 and S3, Supplementary Table S1). Apart from the vertical and horizontal test surfaces V64 and H64 with 64 spots, these differences were statistically significant (ANOVA for comparison between H1, H4, H16 and H64: SS_effect_ = 33407.8, df_effect_ = 3, MS_effect_ = 11135.9, SS_error_ = 17712.9, df_error_ = 24, MS_error_ = 738.0, F = 15.09, p<0.001. ANOVA for comparison between V1, V4, V16 and V64: SS_effect_ = 265210.7, df_effect_ = 3, MS_effect_ = 88403.6, SS_error_ = 383082.3, df_error_ = 24, MS_error_ = 15961.8, F = 5.54, p = 0.005. See also Supplementary Table S2).

For both vertical and horizontal test surfaces the number *N* of trapped tabanids decreased with increasing number and decreasing size of the brown spots: the white vertical test surfaces with 1, 4, 16 and 64 spots captured 67.1%, 31.5%, 1.1% and 0.3% of all tabanids trapped in this experiment, while the same data for the horizontal spotty surfaces were 47.9%, 38.5%, 7.7% and 5.9% (Supplementary Table S1). These differences were again statistically significant (Supplementary Table S2). Test surface V1 captured *Q*(V1/V4) = N_V1_/N_V4_ = 2.1 times more tabanids than test surface V4. The *Q*-values were *Q*(V4/V16) = 28, *Q*(V16/V64) = 3.5, *Q*(H1/H4) = 1.2, *Q*(H4/H16) = 5.0 and *Q*(H16/H64) = 1.3 for the test surface pairs V4/V16, V16/V64, H1/H4, H4/H16 and H16/H64, respectively. The extreme (maximum and minimum) values of *Q* were *Q*(V1/V64) = 208.9 and *Q*(H1/H64) = 8.2 for the test surface pairs V1/V64 and H1/H64, respectively. Test surfaces V64 and H64 with the most numerous and smallest spots were the least attractive, trapping minimal number (V64: 8, H64: 76) of tabanids. The tabanid species flying in the study site during experiments 1 and 2 were: *Tabanus tergestinus*, *T. bromius*, *T. bovinus*, *T. autumnalis*, *Atylotus fulvus*, *A. loewianus*, *A. rusticus*, *Haematopota italica*.

**Figure 2:**
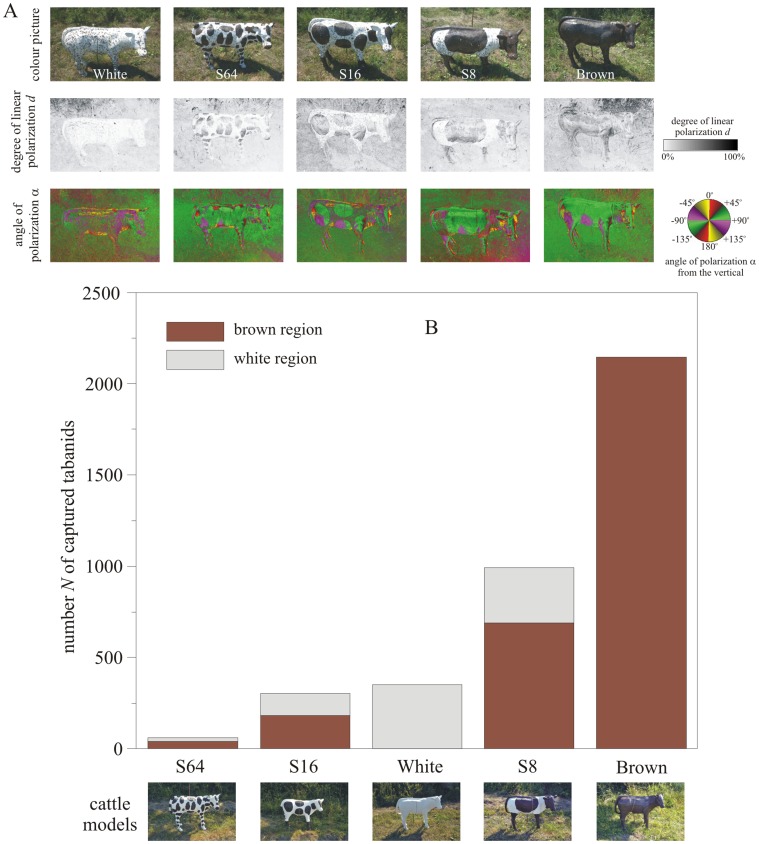
Results of experiment 3. (A) As Fig. 1A for the sticky cattle models used in experiment 3 when the optical axis of the polarimeter was −35° from the horizontal. White: white cattle, S64: white cattle with 64 brown spots, S16: white cattle with 16 brown spots, S8: white cattle with 8 brown spots, Brown: brown cattle. (B, C) Number *N* of tabanids captured by the brown regions (if any) and the white areas of the sticky cattle models in experiment 3.

Similar results were obtained for the sticky cattle models in experiment 3 (Supplementary Table S3, Fig. 2): The homogeneous brown cattle model was the most attractive, trapping 55.63% of tabanids. The cattle model S8 with 8 spots was less attractive (25.76%) than the brown model, but more attractive than the white one (9.15%). The least attractive were the spotty cattle models S16 (7.85%) and S64 (1.61%) with 16 and 64 spots, respectively. The brown spots of cattle models S8, S16 and S64 trapped 2.28, 1.57 and 2.26 times more tabanids than the white surface regions, respectively. Apart from the comparisons between cattle models White and S16 (Supplementary Table S4) as well as between the brown and white regions of cattle model S16 (Table 1), these differences are statistically significant (Table 1 and Supplementary Table S4).

**Table 1:** Statistical results of the ANOVA tests for data in Supplementary Table S3.

compared test surfaces	ANOVA parameters
BM, S8_(B+W), WM, S16_(B+W), S64_(B+W): significant	SS_effect_ = 123210.0, df_effect_ = 4, MS_effect_ = 30802.5, SS_error_ = 123600.5, df_error_ = 110, MS_error_ = 1123.6,F = 27.4, p<0.001
S8_B *versus* S8_W: significant	SS_effect_ = 3272.7, df_effect_ = 1, MS_effect_ = 3272.7, SS_error_ = 22136.3, df_error_ = 44, MS_error_ = 503.1, F = 6.51, p = 0.014
S16_B *versus* S16_W: not significant	SS_effect_ = 97.6, df_effect_ = 1, MS_effect_ = 97.6, SS_error_ = 1591.6, df_error_ = 44, MS_error_ = 36.2, F = 2.7, p = 0.108
S64_B *versus* S64_W: significant	SS_effect_ = 12.5, df_effect_ = 1, MS_effect_ = 12.5, SS_error_ = 47.9, df_error_ = 44, MS_error_ = 1.1, F = 11.5, p<0.001

The spotty cattle models S8, S16 and S64 had a white surface with 8, 16 and 64 brown spots, respectively. BM: brown cattle model, WM: white cattle model, B: brown surface region, W: white surface region.

**Figure 3:**
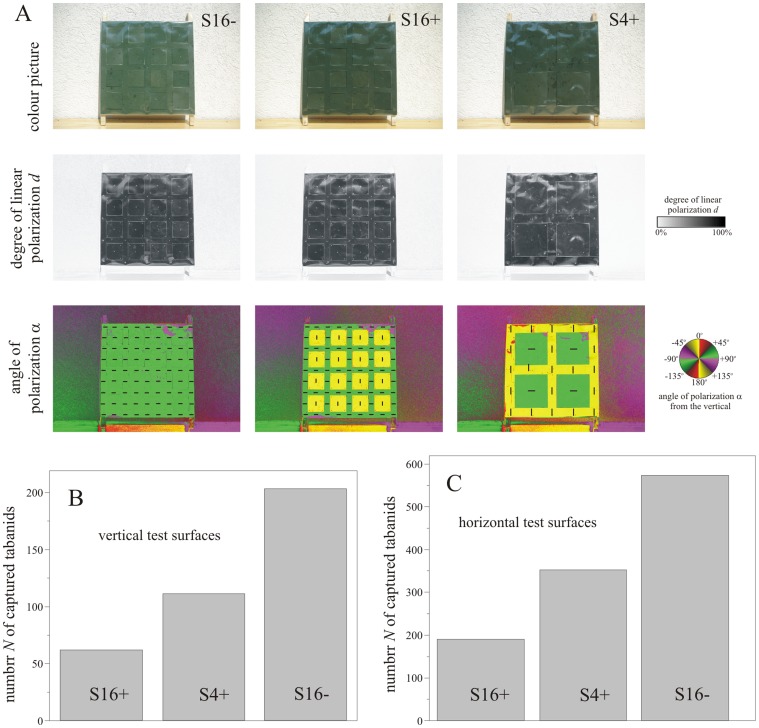
Results of experiment 4. (A) As Fig. 1A for the test surfaces used in experiment 4 when the optical axis of the polarimeter was horizontal. In the α-patterns the short bars represent the local transmission direction of the linear polarizer. (B, C) Number *N* of tabanids captured by the vertical and horizontal test surfaces in experiment 4. S16+: test surface with 16 linearly polarizing squares, the transmission direction of which is perpendicular to that of their surrounding regions. S4+: test surface with 4 linearly polarizing squares, the transmission direction of which is perpendicular to that of their surrounding regions. S16−: test surface with 16 linearly polarizing squares, the transmission direction of which is parallel to that of their surrounding regions.

Figure 3A shows the reflection-polarization patterns of the test surfaces composed of linearly polarizing sheets on matte white substrates used in experiment 4 (Supplementary Fig. S1). In experiment 4 (Supplementary Table S5, Fig. 3, Supplementary Fig. S1) the horizontal and vertical test surfaces H-S16− and V-S16− with homogeneous polarization patterns were the most attractive to tabanids trapping 51.4% and 54.0% of tabanids, respectively. Test surfaces H-S4+ (31.5%) and V-S4+ (29.5%) with 4 spots of different angles of polarization (Fig. 3A) were less attractive, while test surfaces H-S16+ (17.1%) and V-S16+ (16.5%) with 16 angle of polarization spots (Fig. 3A) were the least attractive. The horizontal polarization patterns H-S16−, H-S4+ and H-S16+ attracted more tabanids than the corresponding vertical polarization patterns V-S16+, V-S16− and V-S4+. These differences are again statistically significant (ANOVA for comparison between H-S16−, H-S4+ and H-S16+: SS_effect_ = 3081.9, df_effect_ = 2, MS_effect_ = 1540.9, SS_error_ = 7446.1, df_error_ = 69, MS_error_ = 107.9, F = 14.3, p<0.001. ANOVA for comparison between V-S16−, V-S4+ and V-S16+: SS_effect_ = 427.0, df_effect_ = 2, MS_effect_ = 213.5, SS_error_ = 855.4, df_error_ = 69, MS_error_ = 12.4, F = 17.2, p<0.001. See also Supplementary Table S6).

In our experiments sticky visual targets (2-dimensional surfaces and 3-dimensional cattle models) with varied degrees and angles of polarization of reflected light captured tabanids, the carcasses of which were counted and removed periodically. Each such period was immediately followed by a random re-ordering of the test surfaces. Since the trapped tabanids and other non-tabanid insects were removed, the newly arrived insects were not influenced by the view of insect carcasses. Thus, the altered test surface configuration after each tabanid counting represented a new replication of the given experiment. The number *R* of replications and the minimal (*D*_min_) and maximal (*D*_max_) lengths (days) the repetitions had in our experiments were: experiment 1: *R* = 7, *D*_min_ = 6, *D*_max_ = 12; experiment 2: *R* = 7, *D*_min_ = 6, *D*_max_ = 12; experiment 3: *R* = 23, *D*_min_ = 2, *D*_max_ = 7; experiment 4: *R* = 24, *D*_min_ = 2, *D*_max_ = 8. These numbers of replications were large enough to detect significant differences in the number of trapped tabanids.

**Figure 4:**
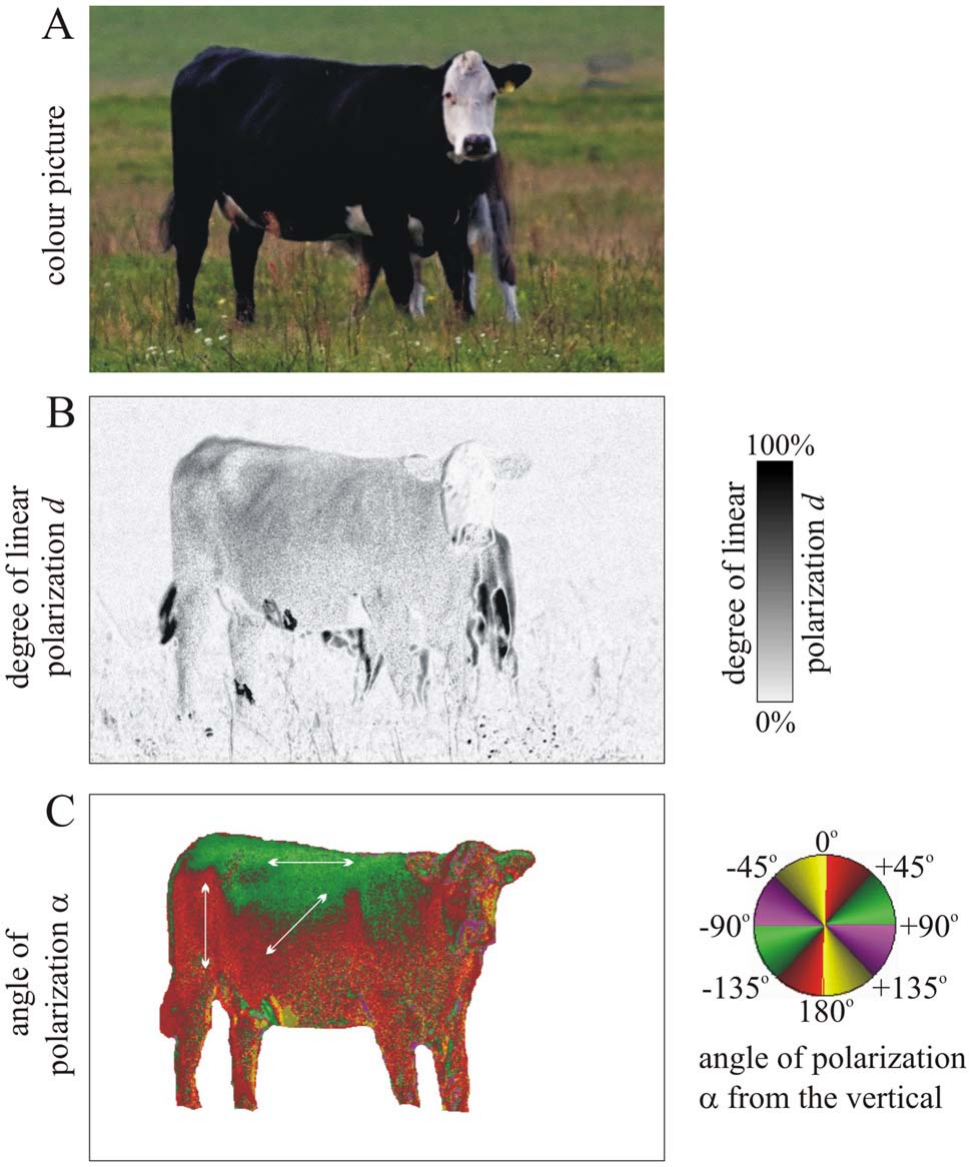
Colour picture and patterns of the degree of linear polarization *d* and angle of polarization *α* (clockwise from the vertical) of a living shady black cattle measured by imaging polarimetry in the blue (450 nm) part of the spectrum. The optical axis of the polarimeter was horizontal, and the measurement was performed under an overcast sky. In the α-pattern double-headed arrows show the angle of polarization of reflected light at some places of the cattle coat. The background of the animal is white for the sake of a better visualization. The body surfaces of the cow from which light is reflected in a vertical plane polarize horizontally, while those from which light is reflected in a horizontal/oblique plane polarize vertically/obliquely.

To compare the reflection-polarization patterns of our spotty test surfaces (Figs. 1A, 2A, 3A) with cattle coats, we measured the reflection-polarization characteristics of a living black cattle (Fig. 4). The coat of black cattle polarizes the reflected light strongly, while the coat of light-coloured cattle polarizes light only moderately or weakly (Supplementary Figs. S4, S5). The neck, back and hindquarters of cattle can reflect light with horizontal polarization (shaded by bright violet and green in Fig. 4 and Supplementary Fig. S4), and other regions of the body surface reflect vertically or obliquely polarized light (shaded by red and yellow in the *α*-patterns of Fig. 4 and Supplementary Fig. S4).

In Supplementary Fig. S4 the reflection-polarization characteristics of a shady (Supplementary Fig. S4A–C) and a sunny (Supplementary Fig. S4D–F) cattle model covered by a white-brown spotty cattle coat are shown from three different directions of view. The brown spots of the cattle coat reflected light with much higher degrees of polarization *d*, than the white regions. Sunlit coats reflected light with higher *d* than shady ones. The backside of the cattle model reflected always horizontally polarized light (encoded by bright violet and green in the *α*-patterns of Supplementary Fig. S4).

In Supplementary Fig. S5 we show how the reflection-polarization patterns of a sunlit horizontal calf coat with white and black spots depend on the viewing direction relative to the solar meridian. The black spots reflected again polarized light with degrees of polarization *d* >60%, while the white spots reflected practically unpolarized light (*d* ≈ 0%). Viewing toward the solar or antisolar meridian, the sunlit horizontal coat reflected always horizontally polarized light. In other viewing directions the angle of polarization of light reflected from the sunny coat was oblique, being always perpendicular to the plane of reflection determined by the sun, the observer and the point observed.

## Discussion

In this work we showed that the spottiness of the coat is of not marginal significance, since it strongly determines the attraction of cattle to tabanids, which has been shown to have severe influence on disease transmission, as well as reduction in milk production and growth due to reduced feeding [14], [17], [18]. On the basis of our results, we suggest that coat colouration could be considered in domestic cattle breeding. According to our study, the brighter the coat and the smaller its dark spots, the less attractive the cattle coat to attacking blood-sucking tabanids. It remains to be studied whether other dipteran insects lured to cattle (e.g. *Musca irritans*) for blood sucking or drinking blood from open wounds are also attracted by the same visual means as tabanids. Many insects have polarization-sensitive eyes [9], and thus may be expected to detect linearly polarized light reflected from dark surfaces including cattle coats.

We show that spots disrupting in intensity and/or angle of polarization the homogeneous pattern of reflected light reduce the attractiveness to tabanids. An appropriately spotty coat pattern can also serve camouflage in a structured optical environment, providing protection against predators [23], [24], [25]. Camouflage seems to be one of the main reasons for spots or stripes in wild animals. There are a number of examples for this theory: Tigers, leopards and many smaller cat species have a striped or spotted coat which makes them hard to detect in the wild [26]. Even an animal as big as a giraffe is well camouflaged by its patterned coat in its natural environment [19].

In this work we present an experimentally supported hypothesis on an earlier unrecognized benefit of spotty coat patterns. We would like to emphasize that unattractiveness to tabanids and other biting insects alone might not explain the evolution of spotty coat patterns in mammals. However, an evolutionary benefit of the spots to the host is a reduced attraction to blood-sucking tabanids. Parasite fauna has been demonstrated to be an important evolutionary force in speciation processes and the evolution of morphological characteristics in a wide range of organisms [27], [28], and may result in a viability cost for the species [29].

In our experiments 1–3, the degree and angle of polarization of light reflected from the test surfaces were confounded with intensity and colour of reflected light, since the brown spots on white background possess both intensity and polarization modulations. Experiment 4, however, was based on utilizing homogeneous dark grey surfaces with quadratic spots of constant degree of polarization varying only in the angle of polarization (Fig. 3A). In this way the important role of polarization in the reduction of the attractiveness of spotty patterns to polarization-sensitive tabanid flies was demonstrated. Experiment 4 was conducted to separate the interaction between intensity (plus degree of polarization) and angle of polarization. The same experimental technique has already been successfully used in a field study showing that tabanids are not attracted to zebra-striped patterns with intensity and/or angle of polarization modulation [20].

Tabanids detect water by means of the horizontal polarization of light reflected from water surfaces [11]. There is also a possibility of polarization affecting their host choice [5]. Recently, a new kind of polarotaxis was observed [13] being governed by the degree of polarization, rather than the angle of polarization of reflected light. They showed that female and male tabanids use horizontal polarization as a cue to find water, while the degree of polarization serves host finding by female tabanids. Thus, tabanids are attracted to linearly polarized light. In our experiments we showed that this positive polarotaxis is gradually reduced by a spotty coat pattern of the host animal as the spot size decreases and the spot number increases. The attractiveness of spotty patterns to tabanids is reduced even if the spots can be perceived only in the angle of polarization pattern. We showed that homogeneous dark grey horizontal and vertical surfaces with spots of orthogonal angles of polarization relative to their surroundings are also less attractive than similar surfaces with a uniform angle of polarization (Fig. 3A). In the case of real spotty coats intensity-colour differences are associated with polarization differences, because bright regions reflect weakly polarized light while dark areas reflect highly polarized light, and the angles of polarization of light reflected from the bright and dark areas are also different. These intensity, colour and polarization differences of spotty patterns synergetically decrease the attractiveness to polarization-sensitive tabanids.

It is important for hematophagous flies to be as cryptic as possible in order to increase their foraging abilities and decrease their predation risk. Flying around or toward larger brown spots could convey an ecological advantage (camouflage) to brown tabanids as they will evade longer the swatting of cattle or the foraging of insectivorous birds.

When flying tabanids are approaching a target with numerous small spots, at first the angular extension of the spots could be lower than the angular resolution (practically coinciding with the interommatidial angle) of tabanid eyes. Thus, from a remote distance the intensity/polarization signal detected would essentially be an average of the light/dark and unpolarized/polarized areas of the target. On the other hand, a homogenous target, or large spots of a spotty target, would be detected as discrete areas from further away. Therefore, one could assume that this would result in certain size assortment by tabanids attracted to targets with different spot sizes: Tabanids with higher visual resolution could be attracted more to spotty targets than tabanids with lower resolution, the latter would only be attracted to targets with larger spots. However, this could be true only for vision from remote distances, because if a tabanid is near to a spotty target, its compound eyes can also perceive the smaller spots.

The host animals of tabanids such as large grazing mammals and cattle emit strong and characteristic odour and breath out carbon-dioxide and aceton. These chemicals surely play a role in the host choice by tabanids [15], [16], [18], [30]. It may be possible that these chemicals are so attractive to tabanids that they would override the visual unattractiveness of spotty coat patterns. It would be an interesting task for future research to study experimentally how odour combines with visual cues from spotty targets in tabanids, and especially the odours emitted by cattle. A similarly important question for future studies would be to analyze whether live cattle with numerous spots are indeed less attractive to tabanids than cattle with less spots. This could be investigated in the field with a similar photographic method as used in [5] to study the attractiveness of living white (grey) and brown (bay) horses to tabanids.

In an earlier study [20] it has been showed that black-and-white striped targets are unattractive to tabanids, and this is an advantage of the zebra stripes. In this work we demonstrated a similar effect, namely the unattractiveness of spotty patterns to tabanids. In both cases, the narrower the stripes and the smaller the spots, the less is their attractiveness to tabanids. This principle could practically be used also for human clothing: wearing appropriately stripy or spotty cloths in areas with large tabanid load, the attraction to blood-sucking female tabanids could be avoided. It would be worth testing this hypothesis in the future.

## Materials and Methods

### Ethics Statement

No specific permits were required for the field studies described here. No specific permissions were required for these locations and activities. The locations are not privately-owned or protected in any way. The field studies did not involve endangered or protected species.

### Experiment 1

Experiment 1 was performed between 10 July and 7 September 2010 in a field near a Hungarian horse farm at Szokolya (47°52′N, 19°00′E), where according to our earlier field experiments [5], several tabanid species were abundant. To test the role of spottiness of vertical test surfaces in the attractiveness to tabanids, four white plastic boards (1 m × 1 m) were set up vertically between metal rods stuck into the ground 5 m apart from each other in the field immediately at the border of a small forest. On both sides of these white boards dark brown, nearly 8-shaped plastic sheets were fixed with glue as spots. The white test surfaces had 1, 4, 16 and 64 such brown spots ordered along a square grid. The ratio of the white and brown areas of the test surfaces was 50∶50%. From 10 July to 5 August the front side (facing toward the field) of the spotty test surfaces was covered by a transparent, colourless, odourless and weather-proof insect monitoring glue (BabolnaBio mouse trap), that captured all insects touching it. All four test surfaces were simultaneously either in the sun or shade. We counted the numbers of tabanids caught by the brown and white regions of the front side of the test surfaces. The glue was refreshed periodically, when the order of the test surfaces was changed randomly to reduce position bias, and the trapped insects with a body size larger than 0.5 mm were also removed from the sticky surfaces. Since the sticky test surfaces were progressively covered by smaller (<0.5 mm) non-tabanid insects producing noise in the experiment, on 6 August the test surfaces were turned, thus their new sticky front sides (facing toward the field) became clean (without disturbing small insects). From 6 August 2010 we counted the numbers of tabanids captured by both the front and back (facing toward the forest) sides of the test surfaces, but the glue was refreshed periodically on the front side only. When the trapped tabanids were removed from the glue, their body suffered so serious damages that their taxonomical identification was impossible. They were, however, identified as tabanid flies (Diptera: Tabanidae) with the use of the taxonomy textbook [17]. Although the sex of the captured tabanids was not determined, from earlier studies [5], [11], [12], [13], [20] we know that vertical and/or elevated test surfaces attract only female tabanids, while horizontally polarizing test surfaces laid on the ground attract female and male tabanids, both being positively polarotactic. In a parallel experiment lasting from 10 July to 7 September 2010, we captured tabanids with a trap composed of a rectangular black plastic tray (50 cm ×50 cm) filled with transparent vegetable oil on the ground. This method of capture made it possible to determine the tabanid species flying in the study site during this experiment.

### Experiment 2

Experiment 2 was performed simultaneously with experiment 1 between 10 July and 7 September 2010 in the same field with the same brown-white spotty and sticky test surfaces, which were laid horizontally onto the ground 100 m apart from the vertical test surfaces used in experiment 1. The aim here was to test the role of spottiness of horizontal test surfaces in the attractiveness to tabanids. To get fresh sticky surfaces, on 6 August 2010 the horizontal test surfaces were turned over: their original lower side faced upward bearing the same sticky and spotty patterns as their other sides. Other details of this experiment were the same as those of experiment 1.

### Experiment 3

Experiment 3 was performed from 22 June to 16 September 2011 at the same site as experiment 1. To test the role of spottiness of 3-dimensional targets in the attractiveness to tabanids, one dark brown, one white and three spotty cattle models (each with the same shape and dimensions; length: 200 cm, height: 120 cm, width: 80 cm) were placed in a normal standing posture along a straight line on the grassy ground and 5 m apart from each other. The cattle models were fixed by a string to a metal rod (1.5 m) stuck into the ground. The spotty models had a white surface with 8, 16 and 64 dark brown spots, the size of which decreased with increasing number. The tabanids trapped by the sticky surface of the cattle models were counted and removed periodically. Other details of this experiment were the same as those of experiment 1.

### Experiment 4

Experiment 4 was conducted between 22 June and 1 September 2011 (Supplementary Fig. S1A) at the same site as experiment 2. To separate the effect of intensity and polarization of light reflected from the target, we tested the role of polarization alone in the attractiveness of spotty targets to tabanids using three different spotty test surfaces (43 cm ×43 cm): S4+ was a surface with 4 neutral grey, linearly polarizing squares (P-W-44, Schneider, Bad-Kreuznach, Germany), the transmission direction of which was perpendicular to that of their surroundings (Supplementary Fig. S1B). S16+ was a surface with 16 linearly polarizing squares, the transmission direction of which was perpendicular to that of their surroundings (Supplementary Fig. S1C). S16− was a surface with 16 linearly polarizing squares, the transmission direction of which was parallel to that of their surroundings (Supplementary Fig. S1D). The intensity and colour of these grey test surfaces were homogeneous. Test surface S16− had also a homogeneous pattern of the degree and the angle of polarization. The degree of polarization reflected both from the 4 and 16 rectangles of test surfaces S4+ and S16+ was the same as that reflected from their surrounding regions, while the angle of polarization of light reflected from these rectangles was perpendicular to that reflected from their surroundings. The substrate of the linearly polarizing sheets was a wooden board (43 cm ×43 cm ×2 cm) painted matte white. The polarizer squares were fixed (with tiny nails) contacting at their margins as tightly as possible on the white substrate. We used one pair of each surface type: the first surface was laid horizontally onto the ground, and the second one was fixed at a height of 1 m above the ground between vertical metal rods stuck into the ground (Supplementary Fig. S1A). The horizontal distance of the horizontal and vertical test surfaces of the same type was 1 m. The three pairs of test surfaces were set along a straight line 5 m apart from each other (Supplementary Fig. S1A). We counted the tabanids trapped by these sticky test surfaces periodically. The intensity and colour (dark grey), furthermore the degree of polarization (*d* ≈ 100%) of the test surfaces were the same, but the angle of polarization varied due to the differing transmission directions of the polarizing squares (Supplementary Figs. S1B–D). Surfaces S4+ (Supplementary Fig. S1B) and S16+ (Supplementary Fig. S1C) presented spotty patterns only in the angle of polarization, while surface S16− (Supplementary Fig. S1D) displayed a homogeneous pattern in the intensity, colour and polarization. At the contacting edges of the polarizing squares there were inevitably narrow gaps. Such gaps occurred in all six test surfaces, because S16−, functioning as a control surface with a homogeneous polarization pattern, had also 16 small linearly polarizing squares cut into the large square. Other details of this experiment were the same as those of experiment 1.

### Measurement of Reflection-Polarization Characteristics

The reflection-polarization characteristics of our test surfaces were measured by imaging polarimetry in the red (650±40 nm = wavelength of maximal sensitivity ± half bandwidth of the CCD detectors of the polarimeter), green (550±40 nm) and blue (450±40 nm) parts of the spectrum. We also measured the polarizing characteristics of a living black cattle, and spotty cattle skins in a Swedish farm in Lund. Our rotating-analyzer, sequential (division of time) imaging polarimeter was a digital camera (Pentax K10), the objective lens of which was mounted with a linear polarizer (PL-CIR HOYA, Japan; diameter: 52 mm) that can be rotated manually. About a given scene three photographs were taken through the polarizer at three different angles (0°, 45°, 90° from the vertical) of its transmission axis. Further details of the method of imaging polarimetry have been described elsewhere [9], [21]. In this work we present only the polarization patterns measured in the blue spectral range, to which tabanids are surely sensitive [15], [22]. Similar patterns were obtained in the red and green parts of the spectrum.

### Statistics

For statistical analyses (ANOVA and binomial χ^2^ test) we used Statistica 7.0.

### Conclusions

From the results of our field experiments presented here we conclude that the spottier a target (host animal) with smaller and more numerous spots, the less attractive it is to tabanids. We also found that the spottier a target of homogeneous intensity, colour and degree of polarization with a pattern of varying angles of polarization, the less attractive is it to polarization-sensitive tabanids. This is an earlier unknown advantage of spotty coats in regions where tabanids are abundant. From our polarization measurements we conclude that the neck, backside and hindquarters of cattle usually reflect horizontally polarized light, while other body parts reflect light with oblique, vertical or horizontal polarization. Furthermore, the darker the coat, the higher the degree of polarization of light reflected from the coat. These reflection-polarization characteristics of the body surface may be general and could be valid for all host animals of tabanids.

## Supporting Information

Figure S1 Photographs of the test surfaces used in experiment 4. (A) Arrangement of the 3 vertical and 3 horizontal sticky test surfaces. (B–D) *Column 1*: Photographs of the test surfaces taken without a polarizer, i.e. as seen with the naked eye. *Column 2*: Photographs of the test surfaces taken through a linear polarizer with a horizontal transmission direction. *Column 3*: Photographs of the test surfaces taken through a linear polarizer with a vertical transmission direction. The double-headed arrows show the transmission direction of the linear polarizer in front of the camera. The short bars represent the local transmission direction of the linear polarizers of the test surfaces. h: horizontal test surface. v: vertical test surface. S4+: test surface with 4 linearly polarizing squares, the transmission direction of which is perpendicular to that of their surrounding regions. S16+: test surface with 16 linearly polarizing squares, the transmission direction of which is perpendicular to that of their surrounding regions. S16−: test surface with 16 linearly polarizing squares, the transmission direction of which is parallel to that of their surrounding regions.
(DOC)

Figure S2 Colour pictures and patterns of the degree *d* and angle α (clockwise from the vertical) of linear polarization of light reflected from the shady brown-and-white spotty vertical sticky test surfaces with 1 (A, H1), 4 (B, H4), 16 (C, H16) and 64 (D, H64) brown spots used in experiment 1 and measured by imaging polarimetry in the blue (450 nm) part of the spectrum from above when the optical axis of the polarimeter was 20° relative to the surface.
(DOC)

Figure S3 As Supplementary Fig. S2 from a side view, when the optical axis of the polarimeter tilted with an azimuth angle of 45° clockwise from the normal vector of the vertical test surfaces.
(DOC)

Figure S4 Reflection-polarization characteristics of a shady (A–C), and a sunny (D–F) cattle coat with white and brown spots measured by imaging polarimetry in the blue (450 nm) part of the spectrum. In D, E, F the polarimeter viewed normally to the solar meridian (NSM), toward the solar meridian (SM), and toward the antisolar meridian (ASM), respectively. The elevation angle of the polarimeter’s optical axis was −20° from the horizontal.
(DOC)

Figure S5 Reflection-polarization characteristics of a sunny horizontal calf coat with white and black spots measured by imaging polarimetry in the blue (450 nm) part of the spectrum from four different directions of view relative to the solar meridian. The elevation angle of the polarimeter’s optical axis was −35° from the horizontal. In the *α*-patterns double-headed arrows show the directions of polarization of reflected light at some places of the coat.
(DOC)

Table S1 Number of tabanid flies (*Tabanus tergestinus*, *T. bromius*, *T. bovinus*, *T. autumnalis*, *Atylotus fulvus*, *A. loewianus*, *A. rusticus*, *Haematopota italica*) trapped by the sticky and spotty test surfaces in experiments 1 and 2 performed between 10 July and 7 September 2010 in a horse farm at Szokolya in Hungary. V: vertical, H: horizontal, B: brown spot, W: white surface region, f: front side of the test surface, b: back side of the test surface. Number of brown spots = 1, 4, 16 and 64 on the test surfaces. The results of statistical tests (ANOVA and χ^2^) can be seen in Table 1 and Supplementary Table S2.
(DOC)

Table S2 Results of the χ^2^ tests for data in Supplementary Table S1. V: vertical, H: horizontal, B: brown spot, W: white surface region; 1, 4, 16, 64: number of brown spots on the test surfaces.
(DOC)

Table S3 Number of tabanid flies (*Tabanus tergestinus*, *T. bromius*, *T. bovinus*, *T. autumnalis*, *Atylotus fulvus*, *A. loewianus*, *A. rusticus*, *Haematopota italica*) trapped by the sticky cattle models in experiment 3 performed between 22 June and 16 September 2011 in a horse farm at Szokolya in Hungary. There were one white, one dark brown and three spotty cattle models. The spotty models had a white surface with 8 (S8), 16 (S16) and 64 (S64) brown spots. B: brown surface region, W: white surface region. The results of statistical tests (ANOVA and χ^2^) can be seen in Table 2 and Supplementary Table S4.
(DOC)

Table S4 Statistical results of the χ^2^ tests for data in Supplementary Table S3. The spotty cattle models S8, S16 and S64 had a white surface with 8, 16 and 64 brown spots, respectively. BM: brown cattle model, WM: white cattle model, B: brown surface region, W: white surface region.
(DOC)

Table S5 Number of tabanids (*Tabanus tergestinus*, *T. bromius*, *T. bovinus*, *T. autumnalis*, *Atylotus fulvus*, *A. loewianus*, *A. rusticus*, *Haematopota italica*) captured in experiment 4 by the horizontal and vertical test surfaces performed between 22 June and 1 September 2011 in a horse farm at Szokolya in Hungary. H: horizontal test surface. V: vertical test surface. S4+: 2×2 = 4 linearly polarizing small squares in a linearly polarizing large square with orthogonal transmission directions. S16+: 4×4 = 16 linearly polarizing small squares in a linearly polarizing large square with orthogonal transmission directions. S16−: 4×4 = 16 linearly polarizing small squares in a linearly polarizing large square with parallel transmission directions. The results of statistical tests (ANOVA and χ^2^) can be seen in Table 3 and Supplementary Table S6.
(DOC)

Table S6 Statistical results of the χ^2^ tests for data in Supplementary Table S5. H: horizontal test surface. V: vertical test surface. S4+: 4 linearly polarizing small squares in a linearly polarizing large square with orthogonal transmission directions. S16+: 16 linearly polarizing small squares in a linearly polarizing large square with orthogonal transmission directions. S16−: 16 linearly polarizing small squares in a linearly polarizing large square with parallel transmission directions.
(DOC)
