# Supplementary material for: Spottier Targets Are Less Attractive to Tabanid Flies: On the Tabanid-Repellency of Spotty Fur Patterns
Source: PLoS One. 2012 Aug 2;7(8):e41138. doi: 10.1371/journal.pone.0041138 (PMC3410892; doi:10.1371/journal.pone.0041138)
Supplement: Figure S1 — Photographs of the test surfaces used in experiment 4. (A) Arrangement of the 3 vertical and 3 horizontal sticky test surfaces. (B–D) Column 1: Photographs of the test surfaces taken without a polarizer, i.e. as seen with the naked eye. Column 2: Photographs of the test surfaces taken through a linear polarizer with a horizontal transmission direction. Column 3: Photographs of the test surfaces taken through a linear polarizer with a vertical transmission direction. The double-headed arrows show the transmission direction of the linear polarizer in front of the camera. The short bars represent the local transmission direction of the linear polarizers of the test surfaces. h: horizontal test surface. v: vertical test surface. S4+: test surface with 4 linearly polarizing squares, the transmission direction of which is perpendicular to that of their surrounding regions. S16+: test surface with 16 linearly polarizing squares, the transmission direction of which is perpendicular to that of their surrounding regions. S16−: test surface with 16 linearly polarizing squares, the transmission direction of which is parallel to that of their surrounding regions. (DOC) [file pone.0041138.s001.doc]

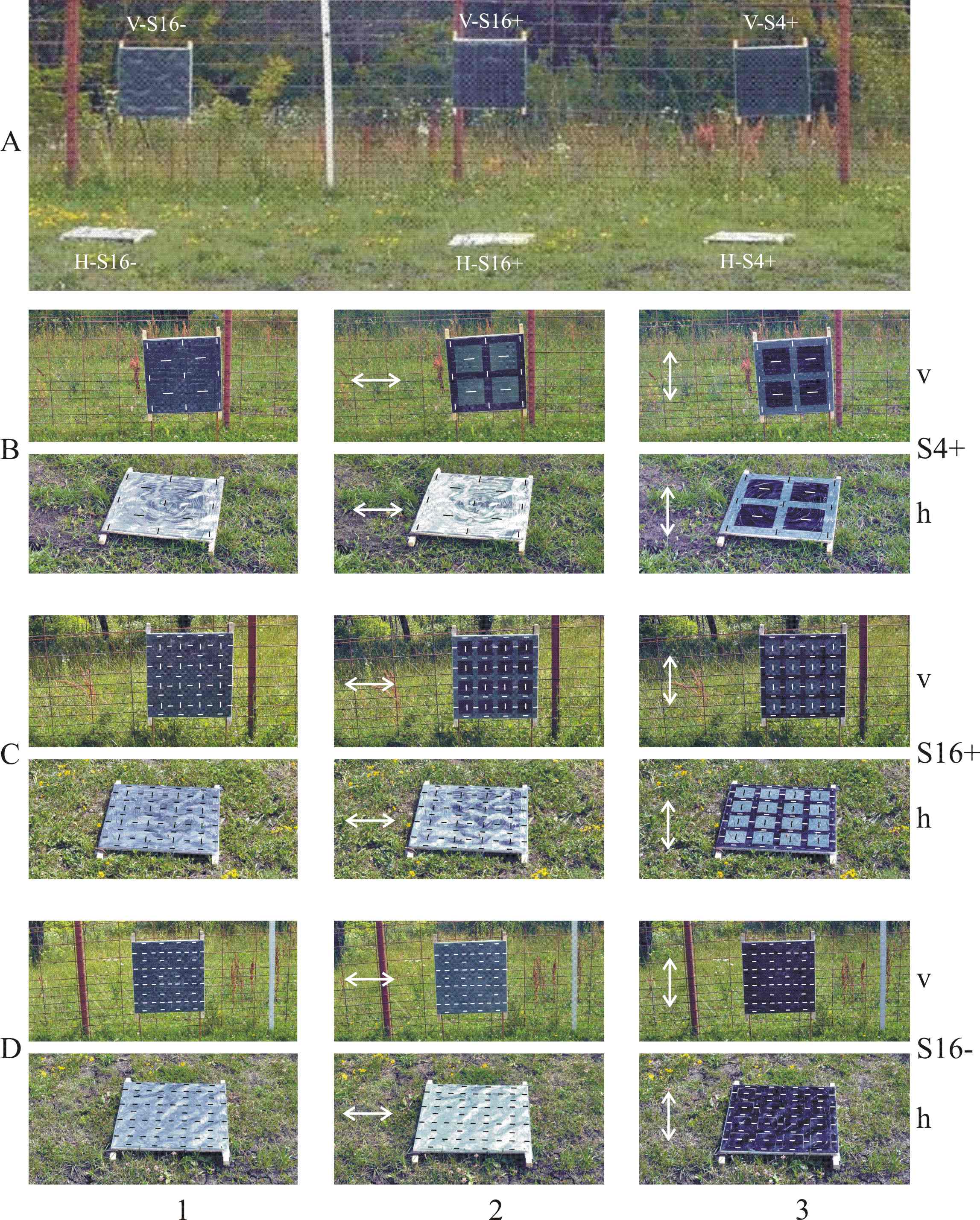


**Supplementary Figure S1**: Photographs of the test surfaces used in experiment 4. (A) Arrangement of the 3 vertical and 3 horizontal sticky test surfaces. (B-D) *Column 1*: Photographs of the test surfaces taken without a polarizer, i.e. as seen with the naked eye. *Column 2*: Photographs of the test surfaces taken through a linear polarizer with a horizontal transmission direction. *Column 3*: Photographs of the test surfaces taken through a linear polarizer with a vertical transmission direction. The double-headed arrows show the transmission direction of the linear polarizer in front of the camera. The short bars represent the local transmission direction of the linear polarizers of the test surfaces. h: horizontal test surface. v: vertical test surface. S4+: test surface with 4 linearly polarizing squares, the transmission direction of which is perpendicular to that of their surrounding regions. S16+: test surface with 16 linearly polarizing squares, the transmission direction of which is perpendicular to that of their surrounding regions. S16−: test surface with 16 linearly polarizing squares, the transmission direction of which is parallel to that of their surrounding regions.
