# Supplementary material for: Spottier Targets Are Less Attractive to Tabanid Flies: On the Tabanid-Repellency of Spotty Fur Patterns
Source: PLoS One. 2012 Aug 2;7(8):e41138. doi: 10.1371/journal.pone.0041138 (PMC3410892; doi:10.1371/journal.pone.0041138)
Supplement: Figure S2 — Colour pictures and patterns of the degree d and angle α (clockwise from the vertical) of linear polarization of light reflected from the shady brown-and-white spotty vertical sticky test surfaces with 1 (A, H1), 4 (B, H4), 16 (C, H16) and 64 (D, H64) brown spots used in experiment 1 and measured by imaging polarimetry in the blue (450 nm) part of the spectrum from above when the optical axis of the polarimeter was 20° relative to the surface. (DOC) [file pone.0041138.s002.doc]

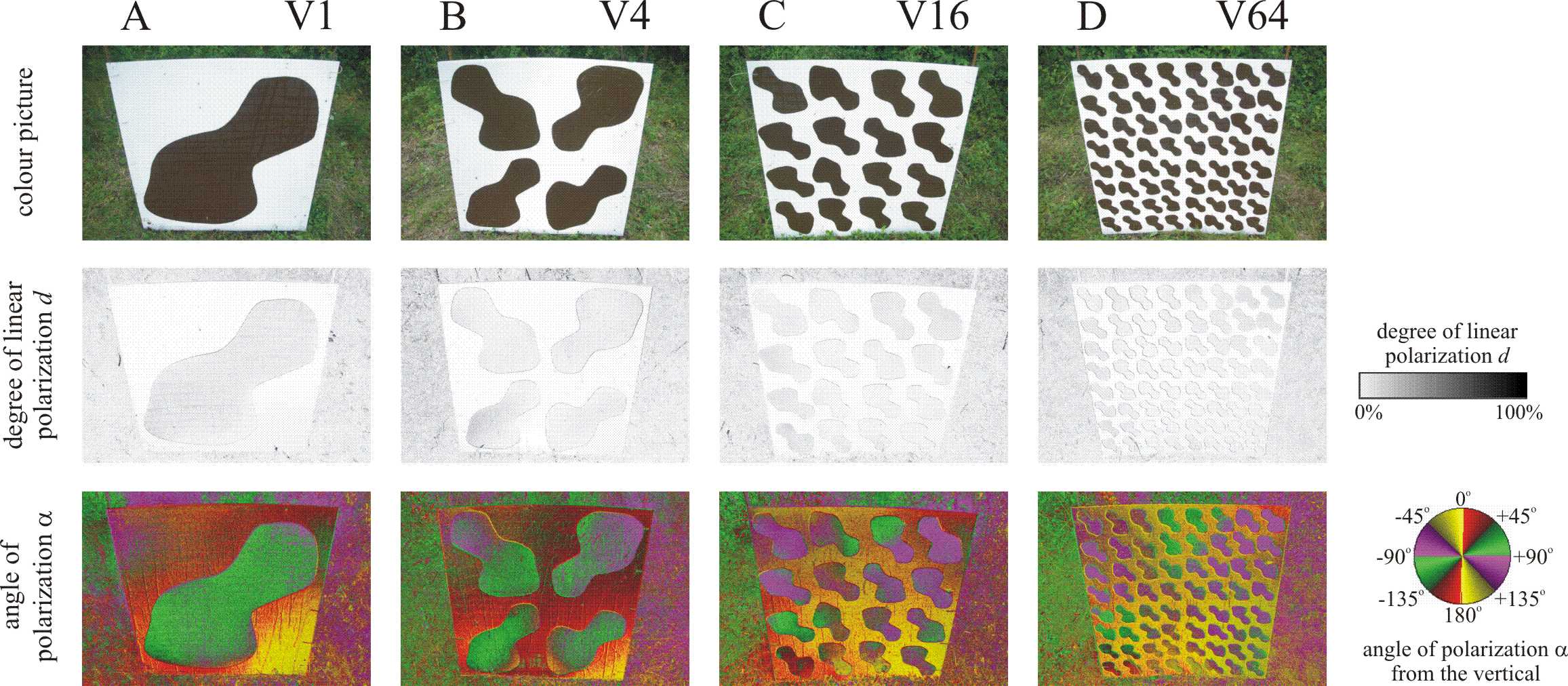


**Supplementary Figure S2**: Colour pictures and patterns of the degree *d* and angle α (clockwise from the vertical) of linear polarization of light reflected from the shady brown-and-white spotty vertical sticky test surfaces with 1 (A, H1), 4 (B, H4), 16 (C, H16) and 64 (D, H64) brown spots used in experiment 1 and measured by imaging polarimetry in the blue (450 nm) part of the spectrum from above when the optical axis of the polarimeter was 20^o^ relative to the surface.
