# Supplementary material for: Spottier Targets Are Less Attractive to Tabanid Flies: On the Tabanid-Repellency of Spotty Fur Patterns
Source: PLoS One. 2012 Aug 2;7(8):e41138. doi: 10.1371/journal.pone.0041138 (PMC3410892; doi:10.1371/journal.pone.0041138)
Supplement: Figure S3 — As Supplementary Fig. S2 from a side view, when the optical axis of the polarimeter tilted with an azimuth angle of 45° clockwise from the normal vector of the vertical test surfaces. (DOC) [file pone.0041138.s003.doc]

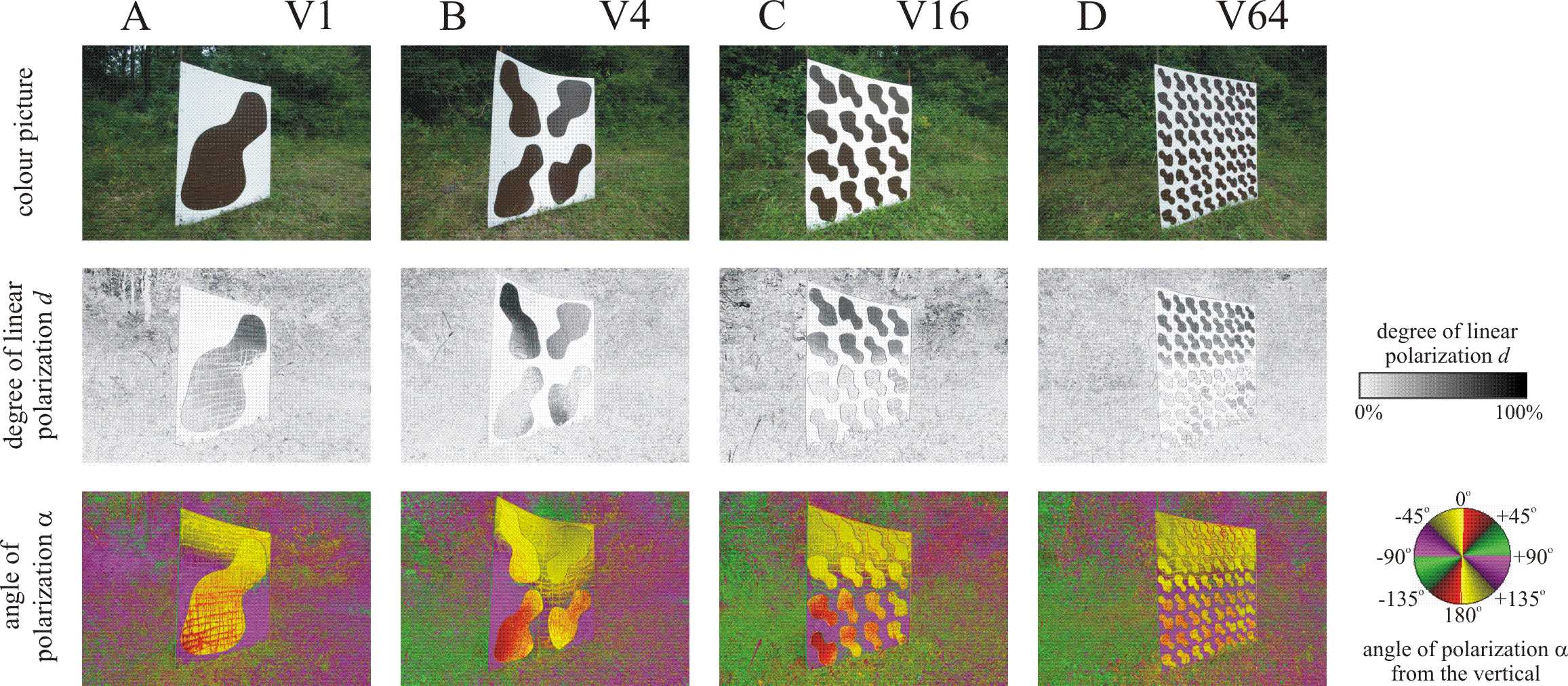


**Supplementary Figure S3**: As Supplementary Fig. S2 from a side view, when the optical axis of the polarimeter tilted with an azimuth angle of 45^o^ clockwise from the normal vector of the vertical test surfaces.
