# Supplementary material for: Spottier Targets Are Less Attractive to Tabanid Flies: On the Tabanid-Repellency of Spotty Fur Patterns
Source: PLoS One. 2012 Aug 2;7(8):e41138. doi: 10.1371/journal.pone.0041138 (PMC3410892; doi:10.1371/journal.pone.0041138)
Supplement: Figure S4 — Reflection-polarization characteristics of a shady (A–C), and a sunny (D–F) cattle coat with white and brown spots measured by imaging polarimetry in the blue (450 nm) part of the spectrum. In D, E, F the polarimeter viewed normally to the solar meridian (NSM), toward the solar meridian (SM), and toward the antisolar meridian (ASM), respectively. The elevation angle of the polarimeter’s optical axis was −20° from the horizontal. (DOC) [file pone.0041138.s004.doc]

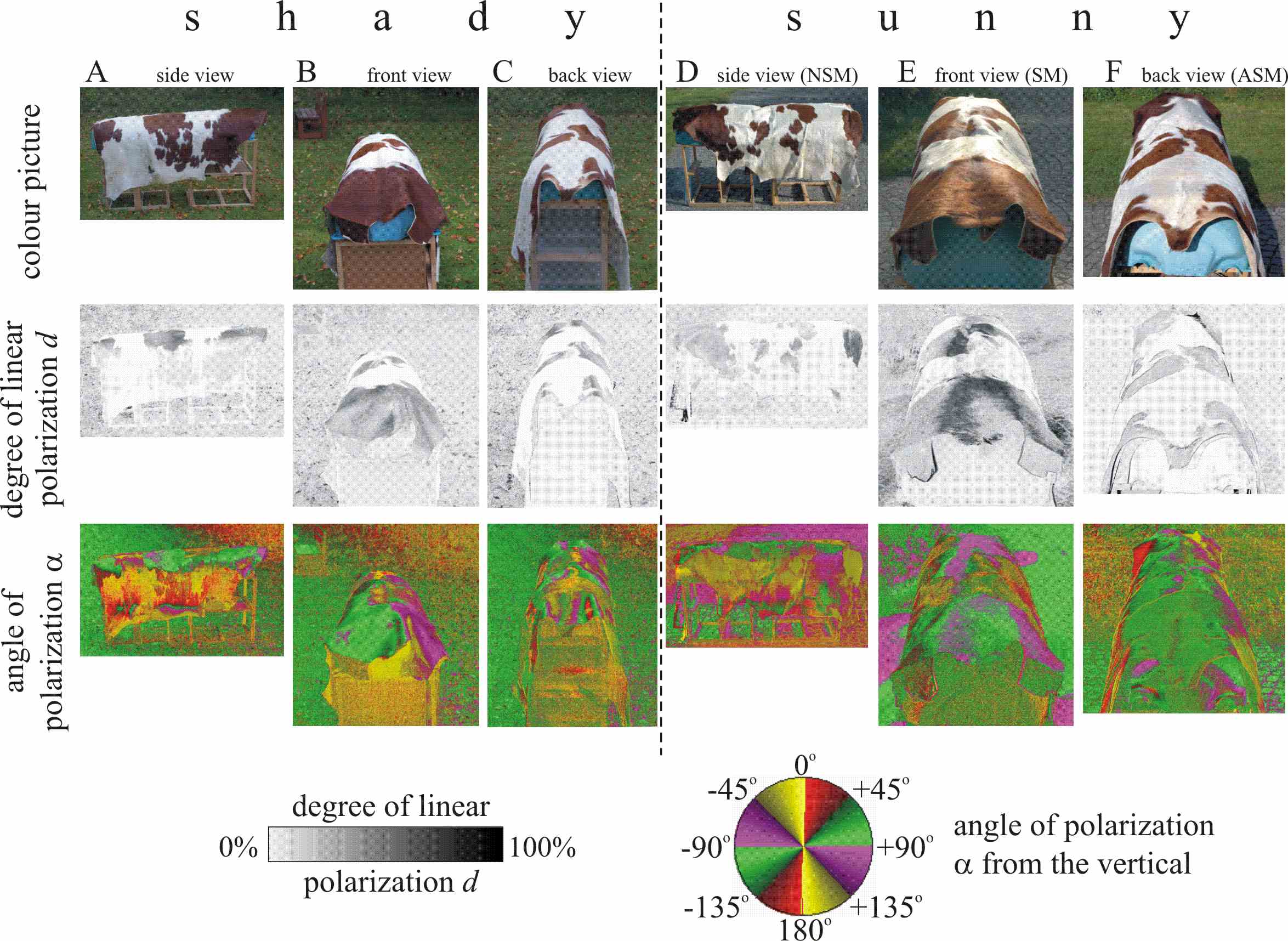


**Supplementary Figure S4**: Reflection-polarization characteristics of a shady (A-C), and a sunny (D-F) cattle coat with white and brown spots measured by imaging polarimetry in the blue (450 nm) part of the spectrum. In D, E, F the polarimeter viewed normally to the solar meridian (NSM), toward the solar meridian (SM), and toward the antisolar meridian (ASM), respectively. The elevation angle of the polarimeter’s optical axis was −20^o^ from the horizontal.
