# Supplementary material for: Spottier Targets Are Less Attractive to Tabanid Flies: On the Tabanid-Repellency of Spotty Fur Patterns
Source: PLoS One. 2012 Aug 2;7(8):e41138. doi: 10.1371/journal.pone.0041138 (PMC3410892; doi:10.1371/journal.pone.0041138)
Supplement: Figure S5 — Reflection-polarization characteristics of a sunny horizontal calf coat with white and black spots measured by imaging polarimetry in the blue (450 nm) part of the spectrum from four different directions of view relative to the solar meridian. The elevation angle of the polarimeter’s optical axis was −35° from the horizontal. In the α-patterns double-headed arrows show the directions of polarization of reflected light at some places of the coat. (DOC) [file pone.0041138.s005.doc]

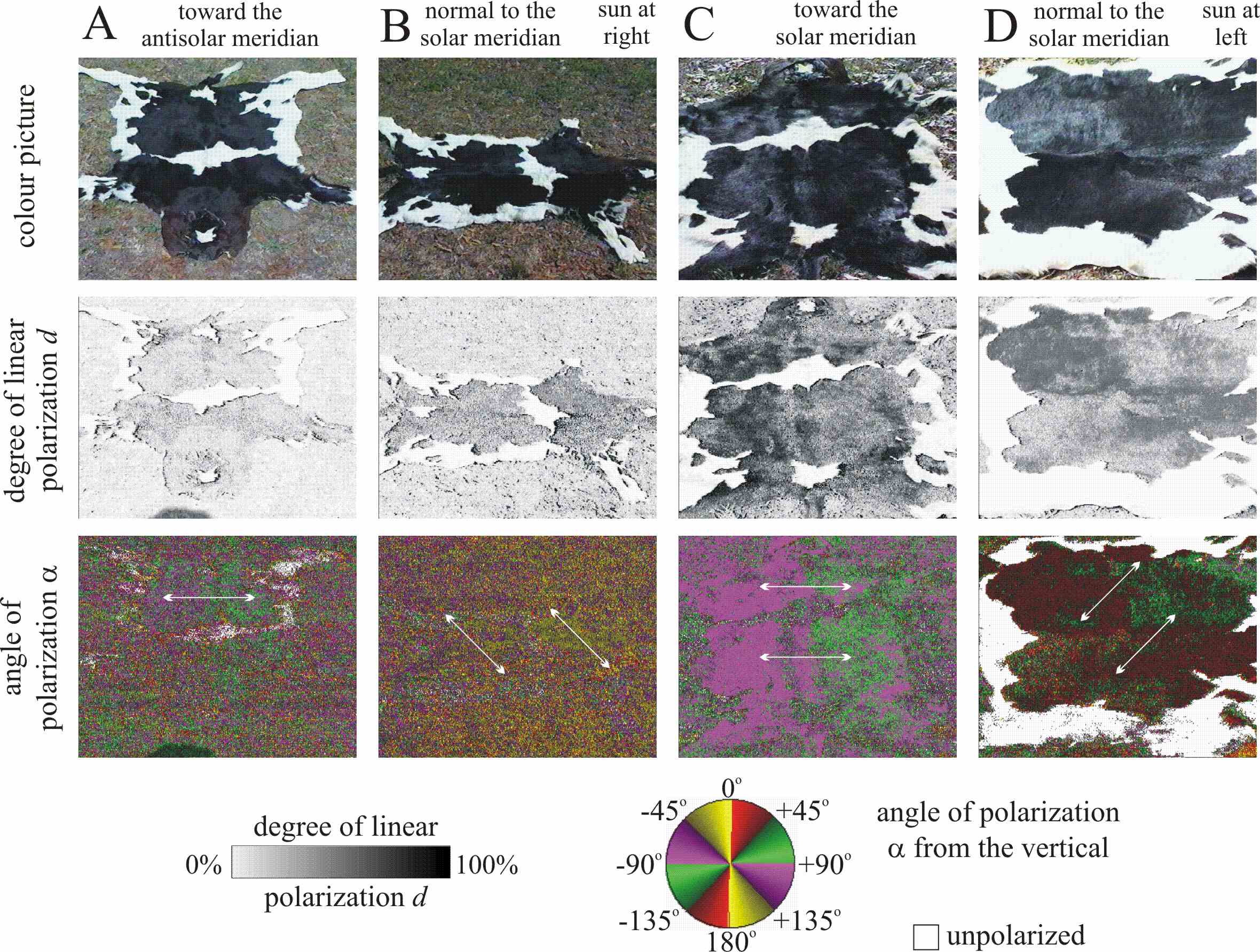


**Supplementary Figure S5**: Reflection-polarization characteristics of a sunny horizontal calf coat with white and black spots measured by imaging polarimetry in the blue (450 nm) part of the spectrum from four different directions of view relative to the solar meridian. The elevation angle of the polarimeter’s optical axis was −35^o^ from the horizontal. In the *α*-patterns double-headed arrows show the directions of polarization of reflected light at some places of the coat.
