# Supplementary material for: Spottier Targets Are Less Attractive to Tabanid Flies: On the Tabanid-Repellency of Spotty Fur Patterns
Source: PLoS One. 2012 Aug 2;7(8):e41138. doi: 10.1371/journal.pone.0041138 (PMC3410892; doi:10.1371/journal.pone.0041138)
Supplement: Table S1 — Number of tabanid flies (Tabanus tergestinus, T. bromius, T. bovinus, T. autumnalis, Atylotus fulvus, A. loewianus, A. rusticus, Haematopota italica) trapped by the sticky and spotty test surfaces in experiments 1 and 2 performed between 10 July and 7 September 2010 in a horse farm at Szokolya in Hungary. V: vertical, H: horizontal, B: brown spot, W: white surface region, f: front side of the test surface, b: back side of the test surface. Number of brown spots = 1, 4, 16 and 64 on the test surfaces. The results of statistical tests (ANOVA and χ2) can be seen in Table 1 and Supplementary Table S2. (DOC) [file pone.0041138.s006.doc]

**Supplementary Table S1**: Number of tabanid flies (*Tabanus tergestinus*, *T. bromius*, *T. bovinus*, *T. autumnalis*, *Atylotus fulvus*, *A. loewianus*, *A. rusticus*, *Haematopota italica*) trapped by the sticky and spotty test surfaces in experiments 1 and 2 performed between 10 July and 7 September 2010 in a horse farm at Szokolya in Hungary. V: vertical, H: horizontal, B: brown spot, W: white surface region, f: front side of the test surface, b: back side of the test surface. Number of brown spots = 1, 4, 16 and 64 on the test surfaces. The results of statistical tests (ANOVA and χ^2^) can be seen in Table 1 and Supplementary Table S2.

| **date (2010)** | **vertical test surfaces (experiment 1)** | | | | **horizontal test surfaces (experiment 2)** | | | |
| --- | --- | --- | --- | --- | --- | --- | --- | --- |
|  | **V1** | **V4** | **V16** | **V64** | **H1** | **H4** | **H16** | **H64** |
| 10-15 July | fB=233, fW=116 | fB=91, fW=63 | fB=2, fW=1 | fB=1, fW=1 | B=93, W=43 | B=61, W=51 | B=10, W=7 | B=5, W=5 |
| 16-22 July | fB=345, fW=201 | fB=174, fW=89 | fB=8, fW=4 | fB=0, fW=2 | B=94, W=43 | B=77, W=45 | B=12, W=1 | B=0, W=4 |
| 23-29 July | fB=293, fW=210 | fB=179, fW=62 | fB=3, fW=1 | fB=1, fW=0 | B=44, W=26 | B=41, W=23 | B=4, W=3 | B=11, W=4 |
| 30 July -  5 August | fB=112, fW=64 | fB=63, fW=22 | fB=5, fW=0 | fB=1, fW=0 | B=66, W=22 | B=44, W=23 | B=6, W=6 | B=8, W=10 |
| **turning the test surfaces** | | | | | | | | |
| 6-17 August | fB=32, fW=15, bB=30, bW=2 | fB=24, fW=1, bB=1, bW=4 | fB=3, fW=0, bB=1, bW=0 | fB=2, fW=0, bB=0, bW=0 | B=59, W=29 | B=57,  W=21 | B=23, W=12 | B=17, W=8 |
| 18-26 August | fB=7, fW=5, bB=4, bW=2 | fB=4, fW=1, bB=5, bW=1 | fB=0, fW=0, bB=0, bW=0 | fB=0, fW=0, bB=0, bW=0 | B=59, W=17 | B=21,  W=23 | B=5, W=5 | B=1, W=0 |
| 27 August -  7 September | fB=0, fW=0, bB=0, bW=0 | fB=0, fW=0, bB=0, bW=0 | fB=0, fW=0, bB=0, bW=0 | fB=0, fW=0, bB=0, bW=0 | B=25, W=5 | B=10, W=6 | B=2, W=5 | B=2, W=1 |
| **sum** | **B=1056, W=615, B+W=1671 (67.1%)** | **B=541, W=243, B+W=784 (31.5%)** | **B=22, W=6, B+W=28 (1.1%)** | **B=5, W=3, B+W=8 (0.3%)** | **B=440, W=185, B+W=625 (47.9%)** | **B=311, W=192, B+W=503 (38.5%)** | **B=62, W=39, B+W=101 (7.7%)** | **B=44, W=32, B+W=76 (5.9%)** |
