# Supplementary material for: Spottier Targets Are Less Attractive to Tabanid Flies: On the Tabanid-Repellency of Spotty Fur Patterns
Source: PLoS One. 2012 Aug 2;7(8):e41138. doi: 10.1371/journal.pone.0041138 (PMC3410892; doi:10.1371/journal.pone.0041138)
Supplement: Table S2 — Results of the χ2 tests for data in Supplementary Table S1. V: vertical, H: horizontal, B: brown spot, W: white surface region; 1, 4, 16, 64: number of brown spots on the test surfaces. (DOC) [file pone.0041138.s007.doc]

**Supplementary Table S2**: Results of the χ^2^ tests for data in Supplementary Table S1. V: vertical, H: horizontal, B: brown spot, W: white surface region; 1, 4, 16, 64: number of brown spots on the test surfaces.

| **compared test surfaces** | **χ^2^** | **df** | **p** | **significance of differences** |
| --- | --- | --- | --- | --- |
| V1_B *versus* V1_W | 116.3 | 1 | < 0.0001 | significant |
| V4_B *versus* V4_W | 113.3 | 1 | < 0.0001 | significant |
| V16_B *versus* V16_W | 9.1 | 1 | 0.0025 | significant |
| V64_B *versus* V64_W | 0.5 | 1 | 0.48 | not significant |
|  | | | | |
| H1_B *versus* H1_W | 104.0 | 1 | < 0.0001 | significant |
| H4_B *versus* H4_W | 25.2 | 1 | < 0.0001 | significant |
| H16_B *versus* H16_W | 5.2 | 1 | 0.0221 | significant |
| H64_B *versus* H64_W | 1.9 | 1 | 0.17 | not significant |
|  | | | | |
| V1_(B+W) *versus* V4_(B+W) | 320.5 | 1 | < 0.0001 | significant |
| V4_(B+W) *versus* V16_(B+W) | 703.9 | 1 | < 0.0001 | significant |
| V16_(B+W) *versus* V64_(B+W) | 11.1 | 1 | 0.0009 | significant |
|  | | | | |
| H1_(B+W) *versus* H4_(B+W) | 13.2 | 1 | 0.0003 | significant |
| H4_(B+W) *versus* H16_(B+W) | 267.6 | 1 | < 0.0001 | significant |
| H16_(B+W) *versus* H64_(B+W) | 3.5 | 1 | 0.06 | not significant |
