# Supplementary material for: Spottier Targets Are Less Attractive to Tabanid Flies: On the Tabanid-Repellency of Spotty Fur Patterns
Source: PLoS One. 2012 Aug 2;7(8):e41138. doi: 10.1371/journal.pone.0041138 (PMC3410892; doi:10.1371/journal.pone.0041138)
Supplement: Table S3 — Number of tabanid flies (Tabanus tergestinus, T. bromius, T. bovinus, T. autumnalis, Atylotus fulvus, A. loewianus, A. rusticus, Haematopota italica) trapped by the sticky cattle models in experiment 3 performed between 22 June and 16 September 2011 in a horse farm at Szokolya in Hungary. There were one white, one dark brown and three spotty cattle models. The spotty models had a white surface with 8 (S8), 16 (S16) and 64 (S64) brown spots. B: brown surface region, W: white surface region. The results of statistical tests (ANOVA and χ2) can be seen in Table 2 and Supplementary Table S4. (DOC) [file pone.0041138.s008.doc]

**Supplementary Table S3**: Number of tabanid flies (*Tabanus tergestinus*, *T. bromius*, *T. bovinus*, *T. autumnalis*, *Atylotus fulvus*, *A. loewianus*, *A. rusticus*, *Haematopota italica*) trapped by the sticky cattle models in experiment 3 performed between 22 June and 16 September 2011 in a horse farm at Szokolya in Hungary. There were one white, one dark brown and three spotty cattle models. The spotty models had a white surface with 8 (S8), 16 (S16) and 64 (S64) brown spots. B: brown surface region, W: white surface region. The results of statistical tests (ANOVA and χ^2^) can be seen in Table 2 and Supplementary Table S4.

| **date (2011)** | **white** | **spotty** | | | **brown** |
| --- | --- | --- | --- | --- | --- |
|  |  | **S64** | **S16** | **S8** |  |
| 25 June | 37 | B = 2, W = 1 | B = 12, W = 4 | B = 29, W = 5 | 50 |
| 27 June | 70 | B = 6, W = 3 | B = 35, W = 18 | B = 141, W = 45 | 235 |
| 29 June | 34 | B = 2, W = 1 | B = 16, W = 10 | B = 64, W = 25 | 166 |
| 2 July | 1 | B = 1, W = 0 | B = 8, W = 3 | B = 7, W = 4 | 58 |
| 6 July | 2 | B = 1, W = 0 | B = 7, W = 3 | B = 12, W = 4 | 61 |
| 10 July | 1 | B = 1, W = 0 | B = 4, W = 2 | B = 8, W = 3 | 39 |
| 14 July | 7 | B = 2, W = 1 | B = 11, W = 11 | B = 40, W = 32 | 119 |
| 18 July | 3 | B = 1, W = 0 | B = 9, W = 5 | B = 20, W = 10 | 80 |
| 21 July | 11 | B = 3, W = 1 | B = 15, W = 13 | B = 50, W = 42 | 214 |
| 24 July | 9 | B = 2, W = 1 | B = 13, W = 11 | B = 48, W = 38 | 190 |
| 28 July | 4 | B = 2, W = 1 | B = 5, W = 4 | B = 40, W = 15 | 126 |
| 31 July | 3 | B = 1, W = 1 | B = 4, W = 3 | B = 23, W = 10 | 101 |
| 3 August | 2 | B = 3, W = 2 | B = 4, W = 3 | B = 38, W = 6 | 41 |
| 8 August | 5 | B = 2, W = 1 | B = 6, W = 4 | B = 21, W = 11 | 63 |
| 11 August | 15 | B = 2, W = 1 | B = 7, W = 5 | B = 22, W = 8 | 107 |
| 17 August | 16 | B = 2, W = 1 | B = 8, W = 5 | B = 20, W = 7 | 95 |
| 21 August | 9 | B = 2, W = 1 | B = 5, W = 3 | B = 10, W = 4 | 40 |
| 26 August | 60 | B = 3, W = 2 | B = 3, W = 2 | B = 17, W = 5 | 101 |
| 29 August | 41 | B = 1, W = 1 | B = 1, W = 0 | B = 8, W = 3 | 86 |
| 1 September | 12 | B = 3, W = 0 | B = 4, W = 3 | B = 20, W = 8 | 65 |
| 4 September | 10 | B = 1, W = 0 | B = 2, W = 1 | B = 13, W = 5 | 50 |
| 9 September | 1 | B = 0, W = 0 | B = 6, W = 4 | B = 33, W = 13 | 41 |
| 16 September | 0 | B = 0, W = 0 | B = 0, W = 1 | B = 7, W = 0 | 18 |
| **sum** | **353 (9.15%)** | **B = 43, W = 19, B + W = 62 (1.61%)** | **B = 185, W = 118, B + W = 303 (7.85%)** | **B = 691, W = 303, B + W = 994 (25.76%)** | **2146 (55.63%)** |
