# Supplementary material for: Spottier Targets Are Less Attractive to Tabanid Flies: On the Tabanid-Repellency of Spotty Fur Patterns
Source: PLoS One. 2012 Aug 2;7(8):e41138. doi: 10.1371/journal.pone.0041138 (PMC3410892; doi:10.1371/journal.pone.0041138)
Supplement: Table S4 — Statistical results of the χ2 tests for data in Supplementary Table S3. The spotty cattle models S8, S16 and S64 had a white surface with 8, 16 and 64 brown spots, respectively. BM: brown cattle model, WM: white cattle model, B: brown surface region, W: white surface region. (DOC) [file pone.0041138.s009.doc]

**Supplementary Table S4**: Statistical results of the χ^2^ tests for data in Supplementary Table S3. The spotty cattle models S8, S16 and S64 had a white surface with 8, 16 and 64 brown spots, respectively. BM: brown cattle model, WM: white cattle model, B: brown surface region, W: white surface region.

| **compared test surfaces** | **χ^2^** | **df** | **p** | **significance of differences** |
| --- | --- | --- | --- | --- |
| BM *versus* S8_(B+W) | 422.6 | 1 | < 0.0001 | significant |
| S8_(B+W) *versus* WM | 305.0 | 1 | < 0.0001 | significant |
| WM *versus* S16_(B+W) | 3.8 | 1 | = 0.051 | not significant |
| S16_(B+W) *versus* S64_(B+W) | 159.1 | 1 | < 0.0001 | significant |
|  | | | | |
| S8_B *versus* S8_W | 151.4 | 1 | < 0.0001 | significant |
| S16_B *versus* S16_W | 14.8 | 1 | = 0.0001 | significant |
| S64_B *versus* S64_W | 9.3 | 1 | = 0.002 | significant |
