# Supplementary material for: Spottier Targets Are Less Attractive to Tabanid Flies: On the Tabanid-Repellency of Spotty Fur Patterns
Source: PLoS One. 2012 Aug 2;7(8):e41138. doi: 10.1371/journal.pone.0041138 (PMC3410892; doi:10.1371/journal.pone.0041138)
Supplement: Table S5 — Number of tabanids (Tabanus tergestinus, T. bromius, T. bovinus, T. autumnalis, Atylotus fulvus, A. loewianus, A. rusticus, Haematopota italica) captured in experiment 4 by the horizontal and vertical test surfaces performed between 22 June and 1 September 2011 in a horse farm at Szokolya in Hungary. H: horizontal test surface. V: vertical test surface. S4+: 2×2 = 4 linearly polarizing small squares in a linearly polarizing large square with orthogonal transmission directions. S16+: 4×4 = 16 linearly polarizing small squares in a linearly polarizing large square with orthogonal transmission directions. S16−: 4×4 = 16 linearly polarizing small squares in a linearly polarizing large square with parallel transmission directions. The results of statistical tests (ANOVA and χ2) can be seen in Table 3 and Supplementary Table S6. (DOC) [file pone.0041138.s010.doc]

**Supplementary Table S5**: Number of tabanids (*Tabanus tergestinus*, *T. bromius*, *T. bovinus*, *T. autumnalis*, *Atylotus fulvus*, *A. loewianus*, *A. rusticus*, *Haematopota italica*) captured in experiment 4 by the horizontal and vertical test surfaces performed between 22 June and 1 September 2011 in a horse farm at Szokolya in Hungary. H: horizontal test surface. V: vertical test surface. S4+: 2 × 2 = 4 linearly polarizing small squares in a linearly polarizing large square with orthogonal transmission directions. S16+: 4 × 4 = 16 linearly polarizing small squares in a linearly polarizing large square with orthogonal transmission directions. S16−: 4 × 4 = 16 linearly polarizing small squares in a linearly polarizing large square with parallel transmission directions. The results of statistical tests (ANOVA and χ^2^) can be seen in Table 3 and Supplementary Table S6.

| **date (2011)** | **horizontal** | | | **vertical** | | |
| --- | --- | --- | --- | --- | --- | --- |
|  | **H-S4+** | **H-S16+** | **H-S16−** | **V-S4+** | **V-S16+** | **V-S16−** |
| 27 June | 13 | 5 | 21 | 5 | 3 | 6 |
| 30 June | 8 | 4 | 16 | 1 | 0 | 2 |
| 4 July | 18 | 10 | 37 | 7 | 3 | 11 |
| 8 July | 3 | 2 | 18 | 2 | 1 | 10 |
| 10 July | 9 | 3 | 14 | 3 | 1 | 7 |
| 12 July | 8 | 3 | 19 | 5 | 3 | 9 |
| 14 July | 0 | 0 | 3 | 0 | 0 | 1 |
| 16 July | 9 | 2 | 15 | 4 | 2 | 8 |
| 18 July | 7 | 4 | 11 | 3 | 1 | 8 |
| 20 July | 10 | 5 | 17 | 6 | 3 | 10 |
| 28 July | 44 | 28 | 53 | 3 | 2 | 5 |
| 5 August | 13 | 8 | 20 | 4 | 3 | 7 |
| 7 August | 15 | 10 | 24 | 6 | 3 | 9 |
| 9 August | 14 | 7 | 21 | 4 | 2 | 6 |
| 11 August | 5 | 2 | 8 | 3 | 1 | 5 |
| 13 August | 7 | 4 | 12 | 1 | 0 | 3 |
| 15 August | 18 | 9 | 30 | 11 | 6 | 11 |
| 17 August | 25 | 14 | 40 | 8 | 5 | 12 |
| 22 August | 26 | 16 | 41 | 5 | 3 | 19 |
| 24 August | 31 | 14 | 43 | 10 | 6 | 19 |
| 26 August | 29 | 17 | 50 | 10 | 9 | 17 |
| 28 August | 17 | 12 | 26 | 3 | 1 | 5 |
| 30 August | 14 | 8 | 20 | 6 | 4 | 10 |
| 1 September | 9 | 4 | 15 | 1 | 0 | 3 |
| **sum** | **352 (31.5%)** | **191 (17.1%)** | **574 (51.4%)** | **111 (29.5%)** | **62 (16.5%)** | **203 (54.0%)** |
