# Supplementary material for: Spottier Targets Are Less Attractive to Tabanid Flies: On the Tabanid-Repellency of Spotty Fur Patterns
Source: PLoS One. 2012 Aug 2;7(8):e41138. doi: 10.1371/journal.pone.0041138 (PMC3410892; doi:10.1371/journal.pone.0041138)
Supplement: Table S6 — Statistical results of the χ2 tests for data in Supplementary Table S5. H: horizontal test surface. V: vertical test surface. S4+: 4 linearly polarizing small squares in a linearly polarizing large square with orthogonal transmission directions. S16+: 16 linearly polarizing small squares in a linearly polarizing large square with orthogonal transmission directions. S16−: 16 linearly polarizing small squares in a linearly polarizing large square with parallel transmission directions. (DOC) [file pone.0041138.s011.doc]

**Supplementary Table S6**: Statistical results of the χ^2^ tests for data in Supplementary Table S5. H: horizontal test surface. V: vertical test surface. S4+: 4 linearly polarizing small squares in a linearly polarizing large square with orthogonal transmission directions. S16+: 16 linearly polarizing small squares in a linearly polarizing large square with orthogonal transmission directions. S16−: 16 linearly polarizing small squares in a linearly polarizing large square with parallel transmission directions.

| **compared test surfaces** | **χ^2^** | **df** | **p** | **significance of differences** |
| --- | --- | --- | --- | --- |
| H-S16− *versus* H-S4+ | 53.2 | 1 | < 0.001 | significant |
| H-S4+ *versus* H-S16+ | 47.7 | 1 | < 0.001 | significant |
|  | | | | |
| V-S16− *versus* V-S4+ | 26.9 | 1 | < 0.001 | significant |
| V-S4+ *versus* V-S16+ | 13.9 | 1 | = 0.002 | significant |
